# Correction: Facile, wafer-scale compatible growth of ZnO nanowires *via* chemical bath deposition: assessment of zinc ion contribution and other limiting factors

**DOI:** 10.1039/d0na90053b

**Published:** 2020-10-23

**Authors:** Yu-Chen Huang, Junze Zhou, Komla Nomenyo, Rodica Elena Ionescu, Anisha Gokarna, Gilles Lerondel

**Affiliations:** Laboratory of Light, Nanomaterials and Nanotechnologies (L2n), CNRS ERL 7004, University of Technology of Troyes 12 rue Marie Curie, BP 2060 10004 Troyes France anisha.gokarna@utt.fr gilles.lerondel@utt.fr

## Abstract

Correction for ‘Facile, wafer-scale compatible growth of ZnO nanowires *via* chemical bath deposition: assessment of zinc ion contribution and other limiting factors’ by Yu-Chen Huang *et al.*, *Nanoscale Adv.*, 2020, DOI: 10.1039/d0na00434k.

The authors regret the omission of a funding acknowledgement in the original article. This acknowledgement is given below.

This research was supported by the ANR research grant (no. ANR-17-CE39-0014).

The Royal Society of Chemistry apologises for these errors and any consequent inconvenience to authors and readers.

## Supplementary Material

